# The beneficial effects of curcumin on aging and age-related diseases: from oxidative stress to antioxidant mechanisms, brain health and apoptosis

**DOI:** 10.3389/fnagi.2025.1533963

**Published:** 2025-01-20

**Authors:** Ying He, Yongqing Liu, Min Zhang

**Affiliations:** ^1^Department of Biological and Food Engineering, Lyuliang University, Lishi, Shanxi, China; ^2^College of Veterinary Medicine, Shanxi Agricultural University, Taigu, Shanxi, China; ^3^Key Laboratory of Agro-Products Primary Processing, Academy of Agricultural Planning and Engineering, MARA, Beijing, China

**Keywords:** aging, age-related diseases, curcumin, signaling pathways, nano-curcumin

## Abstract

Aging and age-related disease are among the most common and challenging issues worldwide. During the aging process, the accumulation of oxidative stress, DNA damage, telomere dysfunction, and other related changes lead to cellular dysfunction and the development of diseases such as neurodegenerative and cardiovascular conditions. Curcumin is a widely-used dietary supplement against various diseases such as cancer, diabetes, cardiovascular diseases and aging. This agent mediates its effects through several mechanisms, including the reduction of reactive oxygen species (ROS) and oxidative stress-induced damage, as well as the modulation of subcellular signaling pathways such as AMPK, AKT/mTOR, and NF-κB. These pathways are involved in cellular senescence and inflammation, and their modulation can improve cell function and help prevent disease. In cancer, Curcumin can induce apoptosis in a variety of different tumor cell lines. Curcumin also activates redox reactions within cells inducing ROS production that leads to the upregulation of apoptosis receptors on the tumor cell membrane. Curcumin can also upregulate the expression and activity of p53 that inhibits tumor cell proliferation and increases apoptosis. Furthermore, curcumin has a potent inhibitory effect on the activity of nuclear factor kappa B *(NF-κB)* and cyclooxygenase-2 *(COX-2)*, which are involved in the overexpression of antiapoptosis genes such as *Bcl-2*. It can also attenuate the regulation of antiapoptosis phosphoinositide 3-kinases (*PI3K*) signaling and increase the expression of *mitogen-activated protein kinases (MAPKs)* to induce endogenous production of ROS. Therefore, herein, we aim to summarize how curcumin affect different epigenetic processes (such as apoptosis and oxidative stress) in order to change aging-related mechanisms. Furthermore, we discuss its roles in age-related diseases, such as Alzheimer, Parkinson, osteoporosis, and cardiovascular diseases.

## Introduction

The process of aging presents a multifaceted challenge that has long captured the attention of the biological sciences. Presently, aging has emerged as a great concern within the field of medicine owing to the substantial surge in the elderly demographic and the concomitant rise in age-associated diseases prevalent in Western societies (Beard et al., 2016). Since the 19th century, the increase of human longevity has been predicated upon advancements in hygiene, medical expertise, and socioeconomic circumstances (Blagosklonny, 2010). In fact, the demographic information indicates a sustained rise in the number of elderly individuals and those in the very elderly category. Individuals aged 65 and older constitute 8.7% of the overall population. Nevertheless, this proportion exhibits regional disparities, with figures approximately ranging from 15 to 16% in North America, Europe, and Central Asia, contrasting starkly with the lower percentage of approximately 5% observed in the Middle East, North Africa, and South Asia (Bielak-Zmijewska et al., 2019). The accumulation of molecular and cellular damage that occur with aging increases the susceptibility of population to a decline in function, diseases, and mortality (López-Otín et al., 2013). Indeed, aging stands as the primary risk factor for numerous chronic and life-threatening human conditions, such as diabetes, osteoporosis, cataracts, Alzheimer’s disease, different types of cancers, cerebrovascular accidents, cardiovascular diseases, and arthritis (Niccoli and Partridge, 2012). Nonetheless, interventions at different levels, including genetic, environmental, and pharmacological fields, have shown promise in mitigating age-related functional decline (Austad and Hoffman, 2021; Kenyon, 2010; Partridge et al., 2020).

Occurrence of multiple age-related diseases in an individual is an important escalating challenge to global healthcare systems. When an individual age, specific biological processes start to show alterations (López-Otín et al., 2013). Collectively, nine aging processes termed “The Hallmarks of Aging” are defined: including cellular senescence, genomic instability, disrupted protein homeostasis, telomere shortening, stem cell exhaustion, epigenetic modifications, impaired mitochondrial function, dysregulated nutrient sensing, and altered intercellular communication. It is noteworthy that these aging hallmarks are not diseases per se but are implicated in the pathogenesis and aberrant physiology of age-related diseases (Aunan et al., 2016). Numerous approaches to mitigating age-related disease have been thoroughly examined, including interventions such as caloric restriction via dietary and exercise control, as well as pharmacological interventions directed at distinct cells and molecules (Di Daniele et al., 2017; Leong, 2018). Despite the notable efficacy demonstrated by these interventions, the administration of medications to the elderly still presents a complex challenge necessitating consideration. This complexity arises from the lack of robust clinical evidence supporting the beneficial outcomes of these pharmaceutical agents. Moreover, side effects are another aspect of these antiaging agents that need more attention and investigation (Li Z. et al., 2021).

Investigations have indicated a variety of signal molecules and pathways that are involved in age-related processes, such as AMP-activated protein kinase (*AMPK*), growth hormone (*GH*)/insulin-like growth factor 1(*IGF1*)/forkhead box O (*FOXO*) pathway, *p38*, mitogen-activated protein kinase (*MAPK*), target of rapamycin (*TOR*)/ribosomal S6 kinase (*S6K*), and sirtuins (*Sirts*) (Kenyon, 2010; Fontana et al., 2010; Haigis and Sinclair, 2010). Apoptosis, or programmed cell death, is another important mechanism occurred during aging process and has two main pathways: intrinsic and extrinsic. The intrinsic pathway is mitochondria-mediated and triggered by internal stressors like DNA damage or oxidative stress (Johnson and Jarvis, 2004; Wu and Bratton, 2013). It involves the activation of pro-apoptotic proteins (e.g., Bax, Bak) that disrupt mitochondrial integrity, releasing cytochrome c to activate caspase-9 and downstream executioner caspases (Johnson and Jarvis, 2004; Wu and Bratton, 2013; Inoue et al., 2009). The extrinsic pathway is initiated by extracellular death ligands (e.g., FasL, TNF-*α*) binding to death receptors, forming the death-inducing signaling complex (DISC), which activates caspase-8. Both pathways converge on executioner caspases (e.g., caspase-3), leading to cellular dismantling while avoiding inflammation, ensuring controlled cell elimination (Yanumula and Cusick, 2020; Ashkenazi, 2008; Nair et al., 2014).

In spite of great endeavors done in order to identify the exact biology of aging and its associated mechanisms at cellular and molecular level, standardized biomarkers and therapeutic agents are still considered inadequate. Indeed, only a few agents that suppress senescence, called senomorphics, and that selectively kill senescent cells, called senolytics, are found (Fuhrmann-Stroissnigg et al., 2017; Hickson et al., 2019; Xu et al., 2018). Natural compounds that are derived from plants have shown great potential in prevention and treatment of a variety of diseases, including age-related diseases (Szymanska et al., 2016). For centuries, these natural products have served as active components in traditional medicine (Dias et al., 2012). The biological and pharmacological properties of certain plant-derived natural products are diverse and significant in contemporary pharmacotherapy. For instance, anticancer agents like paclitaxel, vincristine and vinblastine, and camptothecin, are derived from *Taxus brevifolia*, *Catharanthus roseus*, and *Camptotheca acuminata*, respectively (Atanasov et al., 2015; Khan et al., 2016; Lozano-Pérez et al., 2017).

**Figure 1:**
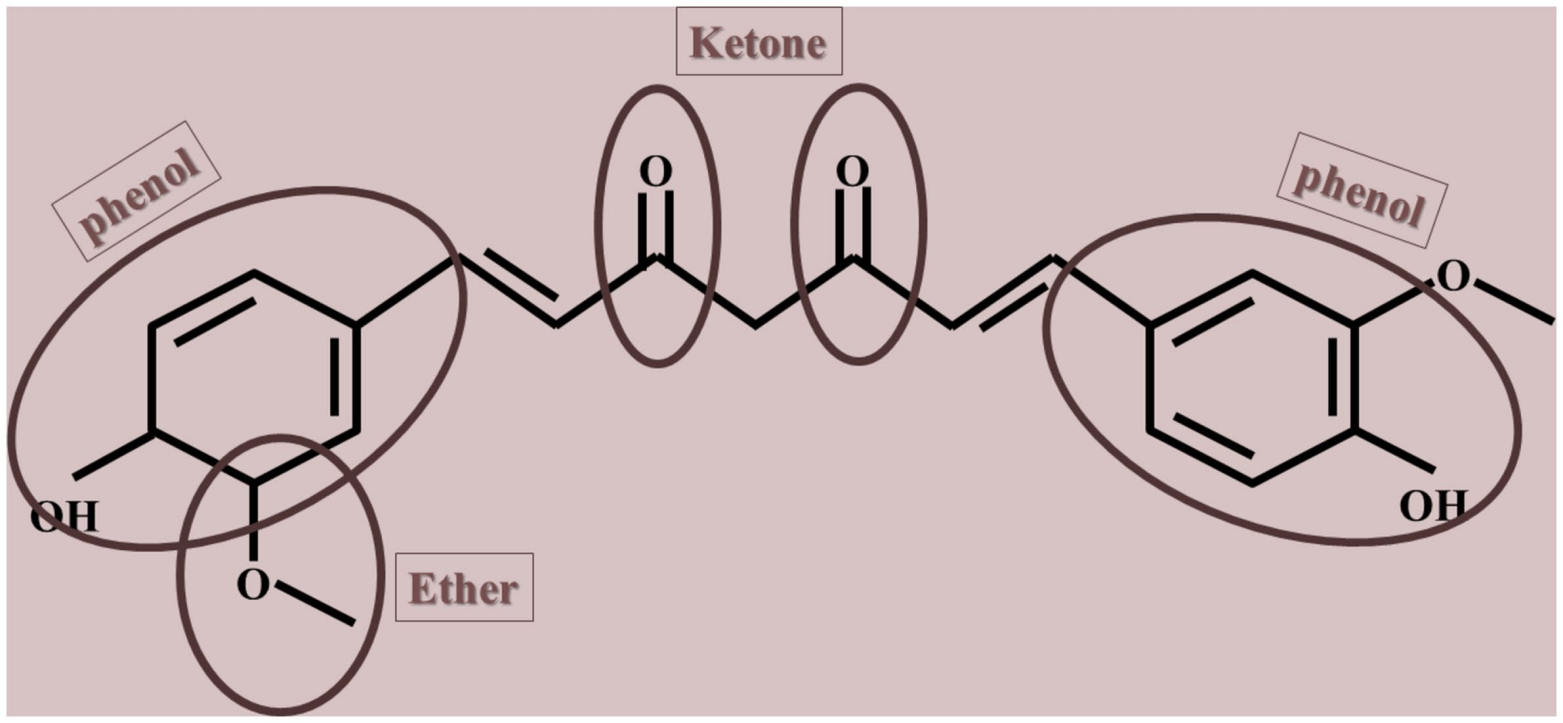
Chemical structure of curcumin.

Natural compounds, such as polyphenols, are considered potential antiaging agents due to their ability to alternate aging hallmarks, including autophagy, oxidative damage, and cell senescence (Vauzour et al., 2010; Singh A. K. et al., 2024; Minocha et al., 2022). Polyphenols have the potential to disrupt a sophisticated array of evolutionarily preserved cellular mechanisms associated with long life spans. Besides, certain polyphenols have attracted interest for their capacity to prolong lifespan in basic organisms and shield against age-related diseases like cancer, cardiovascular diseases, and neurodegenerative conditions. Some of these antiaging agents are omega-3 fatty acids, essential vitamins (i.e., vitamin E, D, K, and C), coenzyme Q10, epigallocatechin gallate, gingerol, collagen, and curcumin (Barcelos and Haas, 2019; Benameur et al., 2021; Chen et al., 2017; Gao et al., 2023; Ozkur et al., 2022; Payne et al., 2022; Thomas, 2006; Troesch et al., 2020). Curcumin, a natural yellow polyphenolic compound derived from the rhizomes of turmeric (*Curcuma longa*) and characterized by the chemical structure 1,7-bis (4-hydroxy-3-methoxyphenyl)-1,6-heptadiene-3,5-dione, is a natural product widely incorporated as a culinary element in various forms of Indian cuisine (shown in Figure 1). The compound is characterized by its lipophilic nature, rendering it insoluble in aqueous environments while exhibiting solubility in ethanol, dimethylsulfoxide, and acetone, with acidity prevailing in acidic and neutral solutions. The extraction of curcumin from turmeric rhizomes is achieved through the utilization of organic solvents (Urošević et al., 2022). Despite its demonstrated therapeutic benefits, several obstacles have limited its clinical utilization, such as low oral bioavailability, poor solubility in aqueous mediums, and susceptibility to degradation in physiological environments. Furthermore, both solid and solution forms of curcumin are sensitive to light. In addition, a significant proportion of curcumin that is ingested orally is eliminated through feces without undergoing metabolic processes, whereas a small fraction that is absorbed into the body undergoes metabolic transformations (Dei Cas and Ghidoni, 2019).

Curcumin exhibits a variety of pharmacological properties which have made it a proper candidate as a preventive or therapeutic agent in numerous health conditions, including but not limited to diseases of pulmonary, autoimmune, renal, and cardiovascular. Furthermore, it is useful in atherosclerosis, different types of cancer, biliary and hepatic disorders, diabetes, wound healing, and chronic inflammation. At cellular and molecular level, curcumin is able to regulate various signaling and signal molecules, such as cytokines, transcription factors, enzymes, growth factors, apoptotic proteins, inflammatory mediators, protein kinases, and cell cycle proteins (Carolina Alves et al., 2019; Leiherer et al., 2013; Prasad et al., 2014). Regarding age-related diseases, curcumin has shown a variety of antiaging effects by modulating the expression of proteins that are involved in aging, such as *AMPK* and sirtuins, while suppressing the expression of proteins that enhance aging, such as *mTOR* and *NF-κB*. Therefore, herein, we discuss how curcumin serve as an antiaging agent that targets a variety of signal molecules and signaling pathways in different age-related diseases.

## Curcumin decelerates aging through affecting oxidative stress

ROS and reactive nitrogen species (RNS) are produced in our cells which have either an endogenous origin such as metabolic pathways (e. g. the tricarboxylic acid (TCA) cycle, the respiratory chain, transport of electrons, and the activity of NADPH) or an exogenous origin (Gomes et al., 2017). Mitochondria have the most essential role in ROS production due to their involvement in many cellular processes containing energy and amino acids metabolism, *β*-oxidation of fatty acids, mitochondrial calcium homeostasis (Gomes et al., 2017; Finkel et al., 2015), heme- and iron–sulfur (Fe-S) cluster biogenesis, regulating cell death, and steroid synthesis. However, mitochondria are not the only cellular component which produce ROS and these free radicals can also develop from cytosol, peroxisomes, nucleus, plasma membrane, endoplasmic reticulum, lysosome (Koutsaliaris et al., 2022). The produced ROS is not always harmful and can be used by cells as a defense mechanism in order to increase cell survival and adaption. However, the amount of ROS and RNS should be kept in a balanced level in order to avoid causing oxidative stress (OS) (Koutsaliaris et al., 2022; Hajam et al., 2022). For this purpose, cells have provided some mechanisms by which gives them the ability to neutralize these free radicals. For instance, superoxide dismutase (SOD) and glutathione peroxidase (GPX) are two main enzymes known for their detoxification effects (Kudryavtseva et al., 2016).

Singlet oxygen (O2), superoxide anion (O2•−), hydrogen peroxide (H2O2), nitric oxide (NO•), hydroxyl radical (OH•), and hydroxyl ion (OH-) are the types of ROS which mitochondria produces and are called mtROS (Kudryavtseva et al., 2016). The process by which mitochondria develops mtROS is started with the construction of a superoxide anion from oxygen by the means of xanthine oxidase (XO) or mitochondrial respiratory chain complexes I (NADH dehydrogenase) and III (bc1 complex) (Li et al., 2013; Papaconstantinou, 2019). The next step of this process is run by SOD which turns these radicals into hydrogen peroxide (Li et al., 2013). Finally, Hydrogen peroxide can be broken down into water and oxygen through different enzymes like glutathione peroxidase, catalase (CAT), or thioredoxin peroxidase (TPx) (Mills, 1960). It can also be converted into hydroxyl radical and hydroxyl ion through a reaction known as the Fenton reaction (Förstermann, 2008). The tight relation between aging and oxidative stress is completely clear and targeting OS has become interesting as an efficient way for inhibiting aging and age-related diseases. As cells age, there is a significant increase in oxidative stress due to a rise in the production of reactive oxygen species (ROS) from dysfunctional mitochondria, coupled with a decline in the body’s ability to neutralize these harmful molecules through antioxidants, leading to damage to cellular components like DNA, proteins, and lipids, which contributes to the aging process itself (Hajam et al., 2022).

The Keap1-Nrf2 pathway is one of the main subcellular signaling pathways involved in antioxidant and detoxification processes (Suzuki and Yamamoto, 2015). It plays a crucial role in maintaining redox homeostasis and protecting cells against oxidative stress, electrophilic damage, and other environmental insults (Yu and Xiao, 2021). The pathway’s primary components are the transcription factor Nrf2 (Nuclear Factor Erythroid 2-Related Factor 2) and its cytoplasmic repressor Keap1 (Kelch-like ECH-associated Protein 1).

In homeostasis, Keap1 binds Nrf2 and promotes its ubiquitination and subsequent degradation by the proteasome (Suzuki et al., 2013). This interaction ensures that Nrf2 levels remain low in the cytoplasm, preventing unnecessary activation of antioxidant response genes (Suzuki and Yamamoto, 2015). Keap1 acts as a sensor for oxidative and electrophilic stress through its reactive cysteine residues (Dinkova-Kostova et al., 2005). When oxidative or electrophilic stress occurs, these cysteine residues undergo modifications that disrupt Keap1’s ability to bind and degrade Nrf2. As a result, stabilized Nrf2 accumulates, translocates to the nucleus, and binds to antioxidant response elements (AREs) in the promoter regions of target genes (Kensler et al., 2007). Nrf2 activation leads to the transcription of a wide range of cytoprotective genes, including those encoding for enzymes like glutathione S-transferases (GSTs), NAD (P) H quinone oxidoreductase 1 (NQO1), and heme oxygenase-1 (HO-1). These proteins play essential roles in neutralizing ROS, detoxifying harmful compounds, and maintaining cellular health (Vomhof-DeKrey and Picklo Sr, 2012; Nguyen et al., 2009).

However, dysregulation of this pathway can have pathological consequences. For instance, while Nrf2 activation is protective against oxidative damage and contributes to cancer prevention, chronic or excessive activation of Nrf2 may support cancer cell survival and proliferation (Wu et al., 2019). Additionally, alterations in this pathway are implicated in neurodegenerative diseases, cardiovascular disorders, and metabolic syndromes (Sivandzade et al., 2019; Zhang et al., 2015). During aging process, the Nrf2/Keap1/ARE signaling pathway becomes less active, and Keap1 and Bach1 levels increase, which represses Nrf2 (Yu and Xiao, 2021).

Curcumin, as an anti-oxidant agent is considered to be a safe way for affecting OS in aged cells through making alterations in different components of OS (summarized in Table 1).

**Table 1:** Studies focusing on the effects of curcumin on reducing oxidative stress in order to decelerate aging process.

Disease/aged organ	Model	Route and dosage of administration	Mechanism(s)	Reference
Aged brain	Animal study	30 mg/kg of curcumin, 5 days/week for 8 weeks, intraperitoneally	Decreasing brain lipid peroxidation and increasing SOD	Cheriki et al. (2024)
	Animal study	200 mg/kg b.w., oral for six weeks	Increasing activity of electron transport chain complexes in the mitochondria	Singh et al. (2024)
	In vitro	5 and 10 μM for 24 h	Suppressing ROS	Jaroonwitchawan et al. (2017)
	In vitro	2.5 μM of curcumin	Suppressing ROS	Lin et al. (2013)
	In vitro	Mono-carbonyl curcumin analogues (h1 − h5)	Suppressing ROS	Hussain et al. (2022)
	In vivo	Mono-carbonyl curcumin analogues (h2 and h3)	Reducing the levels of MDA and enhanced CAT, SOD, and glutathione (GSH) and enhancing memory	Hussain et al. (2022)
	Animal study	Curcumin-loaded nanocapsules (NLC C)	Increasing in the reactive species levels, superoxide dismutase and catalase activities	Fidelis et al. (2019)
	Animal study	BDMC was injected into the lateral ventricles of mice	Enhancing cognition, neurons’ number, SIRT1, and reducing OS	Xu et al. (2020)
Aged heart	Animal study	Curcumin supplemented (0.2%) chow for 4 weeks	Restoring NO-dependent dilation and lowering superoxide levels	Fleenor et al. (2013)
	Animal study	50 mg/kg for 8 weeks		Aziz et al. (2022)
	In vitro	A GMO-based biodegradable nanoparticle (NP) formulation loaded with curcumin	Nanoparticulate curcumin was comparatively more effective than native curcumin in protecting against ROS-induced membrane damage by reducing LPx and LDH leakage	Lim et al. (2001)
Osteoporosis	In vitro	–	Preserving mitochondrial redox potential, decreasing mitochondrial oxidative status, and improving mitochondrial membrane potential and functions.	Dai et al. (2017)
	In vitro	–	Abolishing OS in osteoblasts through inhibiting GSK3β-Nrf2 signaling	Li et al. (2020)

### Curcumin as an antioxidant agent against neurological disorders

Given brain aging and neurodegenerative diseases, curcumin has many protective effects. A study on old female Wistar rats shows that curcumin administration is able to reduce OS in rat’s brain through causing a reduction in brain lipid peroxidation and an induction in SOD levels (Cheriki et al., 2024). In neurodegenerative diseases such as Alzheimer’s disease, one the main risk factors is brain aging as aging increases inflammation and production of oxidative stress while reduces the ability of the cells in production of energy (Floyd and Hensley, 2002; Yin et al., 2016). One of the proposed mechanisms by which curcumin decreases OS in brain is its ability to scavenge free radicals. Curcumin attaches to Fe^2+^ and Fe^3+^ ions, stopping the iron from redox cycling, indicating it might offer another way to lessen Fe2 + −triggered lipid peroxidation (Dairam et al., 2008). Furthermore, comparing high (5,000 ppm) and low doses of curcumin on mice shows that low and high amounts of curcumin effectively decreased oxidized proteins and interleukin-1beta, which is a type of proinflammatory cytokine that is commonly found in the brains of these mice (Lim et al., 2001). When treated with a low amount of curcumin, the astrocytic marker GFAP decreased, while insoluble beta-amyloid (Abeta), soluble Abeta, and plaque burden also decreased by 43–50%. However, the levels of amyloid precursor (APP) in the membrane fraction remained the same. Additionally, the amount of microgliosis in the neuronal layers was suppressed by the curcumin treatment (Lim et al., 2001). Besides curcumin itself, its mono-carbonyl analogues (h1 − h5) have also shown the same effect (somehow even better) on OS and free radical scavenging (Hussain et al., 2022). Both *in vivo* and *in vitro* studies have shown that mono-carbonyl curcumin analogues h2 and h3 with methoxy and chloro-substituents are significantly reducing OS levels (*p* < 0.001) (Hussain et al., 2022). Examining these analogues on mice decreases memory impairment and improves the results of hippocampal-based tests such as light–dark box, hole board, and Y-maze tests. A significant reduction in the levels of *MDA* and enhanced *CAT*, *SOD*, and GSH activities in the hippocampus of mice’s brain is the underlying mechanism by which curcumin analogues enhance memory function (Hussain et al., 2022). Another derivative of curcumin is Bisdemethoxycurcumin (BDMC) which is also examined on AD mice models. After this intervention, Y maze and Morris water maze was used to test the learning and memory ability of mice which both of them were improved due to oxidative stress regulation, a rise in the number of neurons, a reduction in Aβ accumulation, and a boost in *SIRT1* expression.

The most recent study in this field shows that curcumin also works at mitochondria levels to reduce OS in aged cells of brain. In this study, D-gal was used for simulating a condition like aging in male rats’ brains and then these rats were treated with 200 mg/kg of curcumin for 6 weeks. Reverse transcriptase-polymerase chain reaction (RT-PCR) gene expression analysis on these rats showed that OS markers (like *SIRT-1*) were significantly lower after curcumin treatment which was probably the result of increased activity of electron transport chain complexes in the mitochondria of aged brain tissues (Singh A. et al., 2024). Nano-carriers have also recently attracted attention for delivering curcumin in order to increase the bioavailability and biodistribution of this natural compound. Utilizing curcumin-loaded nanocapsules on AD mice model shows that this method is able to decrease the oxidative stress caused by Aβ in the prefrontal cortex, as shown by higher levels of reactive species and increased superoxide dismutase and catalase activities. Notably, NLC-C proved to be more impactful compared to regular curcumin (Fidelis et al., 2019).

Curcumin can also modulate Keap1/Nrf2 pathway and further improves oxidative stress. Curcumin can react with the sulfhydryl group of the Keap1 and further reduce the affinity of Keap1 and Nrf2 which let Nrf 2 to enter the nucleus and further promotes downstream gene targets (Duan et al., 2022). In a study conducted by Duan and colleagues, after induction of intracerebral hemorrhage in rat, they found a significant upregulation of Nrf2 and further restraint of oxidative stress compared to control group (Duan et al., 2022).

Glioblastoma, also known as GBM or glioblastoma multiforme, is a fast-growing, aggressive, and malignant brain tumor that originates in the brain’s glial cells (Wirsching and Weller, 2017). The prevalence of glioblastoma (GBM) in the United States is estimated to be around 3 in 100,000 people. GBM is the most common malignant brain tumor in adults, accounting for about 14% of all primary brain tumors. In the US, more than 12,000 people are diagnosed with GBM each year (Wirsching and Weller, 2017; Grech et al., 2020). Gersey and colleagues found a significant increase of oxidative stress production as well as promotion of MAPK pathway and downregulated STAT3 activity following treatment of glioblastoma cells with curcumin (Gersey et al., 2017).

### Curcumin as an antioxidant agent against cardiovascular disease

Similar to brain, aging has deteriorative effects on cardiovascular system through different mechanisms including increase of oxidative stress, interstitial fibrosis, and reduction of AMPK and GDF11 (Pagan et al., 2022; Derumeaux et al., 2024; Ribeiro et al., 2023). According to studies, one of the ways by which curcumin’s effects can be induced on cardiac aged cells is combining its administration with exercise (Aziz et al., 2022). Assessments on heart tissues of rats who received the combination of these two shows that OS marker SIRT-1 is significantly decreased after this method which might be the result of *MDA* and *NADPH* Oxidase 4 (NOX4) induction. Furthermore, these alterations are more significant when compared to the group which only received curcumin (Aziz et al., 2022). Other than that, using nanotechnology is also another by which curcumin’s effects can be induced on cardiac aged cells. In this regard, Mishra and colleagues provided a glycerol monooleate (GMO)-based biodegradable nanoparticle (NP) formulation and loaded this nanoformulation with curcumin in order to decrease OS *in vitro*. After examining several OS-related markers, findings indicated that curcumin-encapsulated nanoparticles demonstrated superior antioxidant abilities against DPPH and NO radicals compared to regular curcumin at concentrations ranging from 2.5–20 μM. It was also noted that the nanoparticle form of curcumin was more effective than the regular form in preventing membrane damage caused by reactive oxygen species, by reducing LPx and LDH leakage at lower concentrations of 5–10 μM. Additionally, curcumin nanoparticles showed enhanced support for the functions of antioxidant enzymes during the test (Mishra et al., 2024). Heart dysfunction due the accumulation of aged cells in cardiovascular tissues can be also a great target for curcumin’s advantageous effects. Large elastic artery stiffness is one of the factors that might be seen during aging. Part of the reason behind the increasing stiffness of large elastic arteries as we age is due to changes in their structure. These changes involve more collagen I being deposited, less elastin present, and alterations to these proteins caused by AGEs (Fleenor et al., 2013). Fleenor and colleagues used curcumin supplementation on both old and young rats for 4 weeks and assessed aortic pulse wave velocity (aPWV) in these mice as a predictor of elastic artery stiffness and arterial nitrotyrosine abundance as a marker for OS. They observed that the old mice which received curcumin had lower aPWV levels that were similar to those of young control mice (*p* < 0.05) and the amounts of nitrotyrosine was also lower in curcumin treatment group (Fleenor et al., 2013). Curcumin did not have an impact on aPWV in young mice, indicating that curcumin treatment on young cells do not decrease the risks of developing aging. Curcumin supplementation also restored NO-dependent dilation and lowered superoxide levels resulting in ameliorating the impairments in endothelial-dependent dilation to levels not different than young mice (Fleenor et al., 2013).

In atherosclerosis point of view, curcumin decreases lipid peroxidation and thereby, decrease damage in the thoracic and abdominal aorta (Quiles et al., 2002). As well, ROS production can also be decreased in vascular smooth muscle cells. Furthermore, curcumin analogue L3 is also examined on mice model of diabetes in order to find its effects on diabetic atherosclerosis (Zheng et al., 2016). The findings indicated that L3 therapy could lower oxidative stress, boost the function of antioxidants, raise nitric oxide levels in the blood and aortic arch, reduce the creation of harmful oxygen molecules in the pancreas, decrease lectin-like oxidized low-density lipoprotein receptor-1 in the aortic arch, and improve the fatty and buildup in the aortic arch, ultimately stalling the progression of diabetes and its associated issues (Zheng et al., 2016).

### Curcumin as an antioxidant against osteoprosis

In Osteoporosis point of view, a common disease among elderly people, we only found two studies examining curcumin’s effects on reducing OS. One of these studies is conducted by Dai et al. (2017) on an osteoblastic cell line (Saos-2) which were exposed to H2O2 and then mitochondrial ROS and membrane potential were determined using a fluorescence microscope on this cell line. Their assessments showed that curcumin therapy also protected the mitochondria’s ability to maintain balanced redox reactions, reduced oxidative stress within the mitochondria, and enhanced the mitochondria’s membrane potential and efficiency (Dai et al., 2017). It seems that this function of curcumin is possible through causing an induction in the levels of phosphorylated protein kinase B (*Akt*) and phosphorylated glycogen synthase kinase-3β (*GSK3β*) (Dai et al., 2017). Other than that, *GSK3β-Nrf2* signaling is also another way that curcumin works through to reduce OS. Investigating osteoblast dysfunction due to OS indicates that curcumin could effectively counteract oxidative stress, leading to a reduction in the body’s natural production of reactive oxygen species, supporting the survival of bone cells and enhancing the formation of new bone cells. By blocking *GSK3β* and activating *Nrf2*, the damaging impacts of oxidative stress can be significantly minimized, underscoring the importance of the *GSK3β-Nrf2* signaling pathway. Additionally, Curcumin was found to reverse the inhibitory effects of oxidative stress on the *GSK3β-Nrf2* signaling pathway (Li et al., 2020).

### Curcumin as a modulator of subcellular signaling pathways involved in aging process

#### AMPK signaling pathway

*AMPK* is mostly known for its crucial roles in metabolism of eukaryote cells (Mihaylova and Shaw, 2011). The main function of this enzyme is to increase the levels of *ATP* in times of need. *AMPK* is capable of inhibiting anabolism and triggering catabolism when senses the lack or deficit of ATP; when this occurs, this heterodimer binds to adenine nucleotides and gets activated in order to phosphorylate some of key proteins in multiple pathways, including mTOR complex 1 (*mTORC1*) and as well, make some alterations in lipid homeostasis, glycolysis, and mitochondrial homeostasis (Herzig and Shaw, 2018). AMPK, itself, is composed of three components: a regulatory beta unit, a catalytic alpha unit, and another regulatory unit name gamma. These subunits are encoded by different genes such as *PRKAA1, PRKAB1,* and *PRKAG1* (Mihaylova and Shaw, 2011; Herzig and Shaw, 2018). As a matter of fact, this signaling pathway is confirmed to participate in aging process and causing age-related diseases (Stancu, 2015). When energy levels are low, AMPK is activated, which can slow aging and increase resistance to age-related diseases. AMPK activation can also help with cellular homeostasis, metabolism, and stress response (Salminen and Kaarniranta, 2012).

### Curcumin and diabetes mellitus: role of AMPK pathway

Type 2 diabetes is one of the most known and studied age-related diseases as aging leads to the insulin resistance due to the change of the body composition by aging as well as increase of the risk of age-related disease such as cardiovascular disease and kidney disease which pose challenges to diabetes management (Committee, 2023; Huang et al., 2014; Ryan, 2000; Boden et al., 1993). Increase of OS during progression of diabetes mellitus is one of the well-known mechanisms involved in this disease (Caturano et al., 2023). This disease can be targeted by curcumin through regulating many fundamentals of AMPK signaling pathway (also summarized in Table 2). Curcumin not only increases the expression and phosphorylation of *AMPK* itself but also affects other related proteins such as acetyl-CoA carboxylase (ACC) and thereby, decreasing blood glucose 400 times better than metformin (Jiménez-Flores et al., 2014; Kim et al., 2009; Lu et al., 2019). Besides, curcumin is also able to decrease the complications of diabetes in elderlies. For instance, diabetic nephropathy is one of these unwanted consequences of diabetes which can be inhibited by curcumin (Kim et al., 2016). Curcumin activates *AMPK* signaling which leads to *Nrf2* induction and decreasing OS and thus, it exerts its reno-protective effects by inhibiting renal lipid accumulation (Kim et al., 2016). diabetic encephalopathy (DE) is also another complication that curcumin has the ability to reduce in diabetic rats through the down-regulation of AMPK-mediated gluconeogenesis (Lakshmanan et al., 2011). Regarding the other complication of diabetes. Curcumin stimulated *AMPK* and *JNK1*, leading to the phosphorylation of Bcl-2 and Bim. This caused them to separate from *Beclin1*, promoting autophagy and reducing apoptosis. Furthermore, curcumin likely regulates autophagy by inhibiting the *mTORC1* pathway through *AMPK* in diabetic conditions. Their research indicates that curcumin can defend against diabetic cardiomyopathy by controlling the interaction between autophagy and apoptosis (Yao et al., 2018).

**Table 2:** Curcumin’s effects on activating AMPK pathway for inhibiting aging.

Age-related disease	Model	Curcumin dosage/administration	Mechanism(s) of action	Reference
Diabetes	Animal	0.75% curcumin mixed with diet for 8 weeks	Increasing the expression of AMPK	Jiménez-Flores et al. (2014)
	In vitro	–	Increasing the phosphorylation of AMPK and ACC and suppressing hepatic gluconeogenesis	Kim et al. (2009)
	Animal	50 and 100 mg/kg, orally gavage every day	Enhanced AMPK activation	Lu et al. (2019)
	Animal	100 mg/kg/day	Decreasing albuminuria, urinary MDA, and SOD and serum lipid-related index and ectopic lipid accumulation through activation of AMPK/Nrf2 signaling	Kim et al. (2016)
	Animal	Orally administered curcumin (100 mg/kg BW) for 8 weeks	Upregulating protein expression of p-AMPKα1 and down-regulation of AMPK-mediated gluconeogenesis	Lakshmanan et al. (2011)
	Animal	Orally, 200 mg/kg/day for 3 months	Activating AMPK and JNK1, which phosphorylated Bcl-2 and Bim and thereby promoting autophagy and alleviating apoptosis	Yao et al. (2018)
Cerebrovascular dysfunction	Animal	Dietary curcumin (0.2%) for one month	Reducing OS through AMPK activation in cerebral arteries	Pu et al. (2013)
	In vivo	–	Preventing Neuroinflammation by inducing Microglia to transform into the M2-phenotype via CaMKKβ-dependent activation of the AMPK Signal Pathway	Qiao et al. (2020)
Cardiovascular diseases	In vitro	–	Protecting cardiomyocytes from apoptosis through the AMPK pathway and regulate Bax and Bcl-2.	Zhang et al. (2019)
	In vitro	–	Promoting autophagy by enhancing the SIRT1/AMPK/mTOR pathway	Yang et al. (2022)

### Curcumin against cerebro- and cardiovascular disease: role of AMPK pathway

Investigations on aging-related cerebrovascular dysfunction (including cerebral amyloid angiopathy, cognitive decline and neurodegenerative diseases) have highlighted other beneficial roles for this natural compound. Curcumin dietary administration on 24-month-old male rodents shows that 1 month of giving curcumin greatly improves the damaged ability of the blood vessels of brain to relax in old rats. In the brain arteries of these rats and in cultured endothelial cells, curcumin increases the phosphorylation of eNOS and AMPK, and reduces the production of ROS. These benefits from curcumin were lost when AMPK was blocked; as a result, curcumin reduces OS through AMPK activation in cerebral arteries (Pu et al., 2013). Decreasing neuro-inflammation through AMPK activation in BV2 microglial cells probably by the means of two of upstream kinases of AMPK (Calmodulin-dependent protein kinase kinase *β* (*CaMKKβ*) and liver kinase B1 (*LKB1*)) (Qiao et al., 2020). This effect of curcumin is mostly beneficial for inhibiting neurodegenerative diseases than cerebrovascular accidents. Regarding aged-cardiac cells, we only found two studies which confirmed AMPK activation after curcumin administration. Zhang et al. (2019) created nanoparticles with optimal conditions, resulting in a high drug content and small size of particles. They declared that one possible way that curcumin NPs work is by activating a specific signaling pathway involving AMPK that may regulate the expression of downstream proteins like Bax and Bcl-2 in palmitate-treated H9C2 cells and thereby, protect cardiomyocytes from apoptosis (Zhang et al., 2019). Another study on senescent cardiomyocytes shows that curcumin can also affect these cells through inhibiting phosphorylation of mTOR and thereby, activating AMPK in a dose-dependent manner (Yang et al., 2022).

#### Akt/PI3K/mTOR signaling pathway

The *PI3K/protein kinase B (AKT)/mTOR* signaling pathway is famous for its key roles in cell cycle and growth regulation. A summarized version of this signaling is that *PI3K* modifies phosphatidylinositol-4,5-bisphosphate (*PIP2*) to produce phosphatidylinositol-3,4,5-trisphosphate (PIP3), which attracts cancer-causing signaling proteins, such as the enzyme Akt. Once activated, Akt alters various targets. *mTOR*, a frequent target of Akt’s actions, is one of the main proteins affected (He et al., 2021). However, there are some other downstream proteins such as *FOXO, Bad, mdm2*, and *GSK3*.

As an age-related disease, cancer is widely studied regarding the pivotal effects of this signaling in angiogenesis, cell proliferation, metastasis, and etc. Telomere dysfunction, DNA repair deficiencies, and increased likelihood of DNA and protein damage during the aging process are among the main mechanisms linking aging to cancer (Anisimov, 2007; Havas et al., 2022; Sedrak and Cohen, 2023).

**Figure 2:**
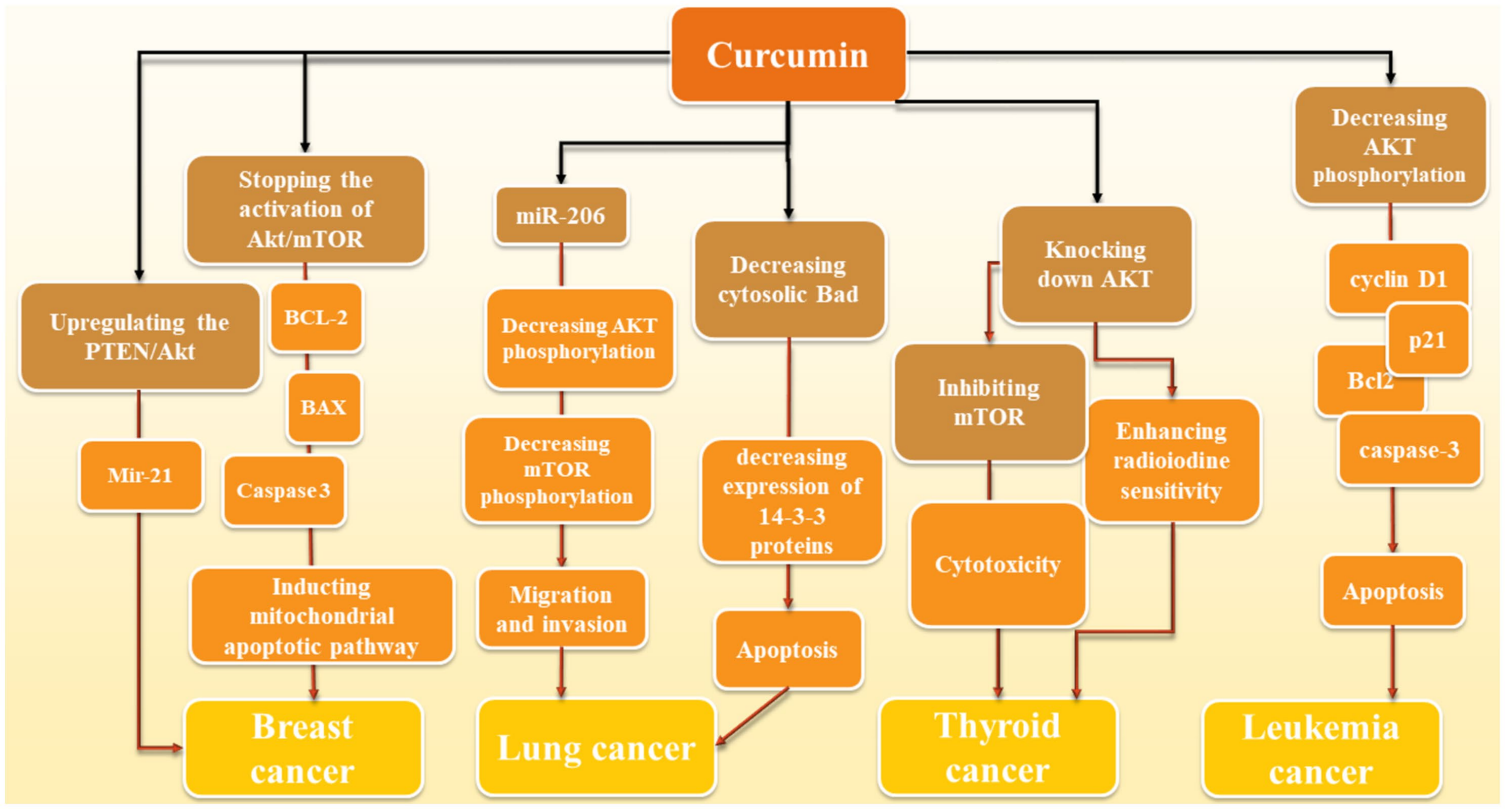
Molecular action of curcumin in various cancers: Curcumin mediates its effects through different mechanisms. In breast cancer, via upregulation of PTEN/Akt and further regulation of miR-21, it inhibits this cancer progression. Moreover, inhibition of Akt/mTOR and further induction of mitochondrial apoptotic pathway through BCL-2, BAX, Caspase-3 is another proposed mechanism of curcumin against breast cancer. Elevation of miR-206 and further reduction of Akt/mTOR (similar to breast cancer) and reduction of cytosolic Bad and further reduction of 14–3-3 proteins and induction of apoptosis are two mechanisms in which curcumin has a significant role in reduction of lung cancer development. In thyroid cancer, reduction of Akt by curcumin leads to the increase of cancer cells to radiotherapy and inhibition of mTOR mediates the cytotoxic effects of curcumin in this cancer. Decrease of Akt phosphorylation also induces apoptosis via modulation of apoptotic proteins such as caspase-3 and Bcl-2 as well as p21 and cyclin D1.

Curcumin can inhibit cancer progression through Akt/PI3K/mTOR signaling pathway; however, due to wide range of cancers and curcumin’s effects we were not able to mention all the studies and the section below is just giving some examples (also summarized in Figure 2).

## Leukemia

In leukemia point of view, an *in vitro* study shows that 25 μΜ curcumin for 24 h treatment reduces the activation of proteins like *AKT, PRAS40, 4E-BP1, P70S6K, RAF-1*, and *p27* in AML cell lines in a dosage-dependent manner. It also had an impact on cell cycle and apoptosis-related proteins *cyclin D1, p21, Bcl2*, cleaved-caspase-3, and cleaved-PARP, causing cell cycle arrest and apoptosis in these cell lines. The effects were stronger when combined with the *AKT* inhibitor afuresertib but weakened by the *AKT* activator SC-79 which confirms that all these effects are AKT-dependent (Zhou et al., 2021). Another similar study tested doses from 0 to 80 μM of curcumin on 697, REH, SupB15, and RS4; 11 cells and found out curcumin treatment induces a dephosphorylation of the constitutive phosphorylated *AKT/PKB* which leads to suppressing cancer cell growth and apoptosis induction (Kuttikrishnan et al., 2019). However, we did not find any human studies in this field.

## Lung cancer

In regards to lung cancer, curcumin exerts its effect through the reduction of levels of 14-3-3 proteins, which leads to a decrease in cytosolic Bad protein and an increase in mitochondrial Bad. This resulted in a lowering of the mitochondrial membrane potential. 14-3-3 proteins typically bind to Bad that has been phosphorylated by *AKT*, stopping it from moving to the mitochondria where it could cause cell death. Curcumin not only lowered the amount of 14-3-3 proteins but also encouraged Bad to be dephosphorylated in a way that depended on *AKT* (Endo et al., 2020). Curcumin was also found to greatly reduce the movement and penetration of A549 cells. This was linked to a noticeable increase in the levels of miR-206 expression. When miR-206 was overexpressed, it hindered the movement and penetration of A549 cells; however, it also notably lowered the levels of *mTOR* and *AKT* phosphorylation. On the contrary, when miR-206 was inhibited, it encouraged cell movement and penetration while increasing *mTOR* and *AKT* phosphorylation. Additionally, introducing miR-206 mimics strengthened curcumin’s ability to inhibit cell movement (Wang et al., 2020). Furthermore, the outcomes of another study demonstrated that curcumin could reduce the viability of human non-small cell lung cancer (NSCLC) cells in a manner that depended on both the duration and amount of dosage. Additionally, after treatment with curcumin, there was a noticeable increase in autophagy, as evidenced by the presence of fluorescent particles (autophagic vesicles) and a significant rise in the *LC3-II/LC3-I* and Beclin1 ratio, along with a decrease in p62 expression. Additionally, it was found that curcumin greatly decreased the activity of the *PI3K/Akt/mTOR* pathway. Interestingly, combining curcumin with inhibitors of *mTOR* or *PI3K/Akt* further enhanced cell death and self-degradation triggered by curcumin, resulting in a notable reduction in cell growth (Liu et al., 2018). According to the results of another study, curcumin is also able to affect epithelial-mesenchymal transition (EMT) along with migration by the means of blocking HGF-induced c-Met phosphorylation and downstream activation of *Akt, mTOR*, and *S6* (Jiao et al., 2016).

## Thyroid cancer

In thyroid cancer cells curcumin is effective for decreasing drug resistance. Radioactive iodine (RAI) is a frequently used treatment for differentiated thyroid cancer (DTC). A significant obstacle is when DTC loses its ability to absorb radioiodine. Patients whose cancer has spread to distant areas often face ongoing disease and resistance to RAI treatment because their tumors have become less responsive. As a result, the idea of re-differentiating tumors to regain sensitivity to RAI therapy is seen as a potential solution to overcome this resistance (Zhang et al., 2021). Curcumin improves the processing and transportation of NIS on cell membranes, leading to a notable increase in radioiodine absorption in laboratory tests. Knocking down *AKT* showed similar effects to restoring thyroid-specific gene expression. On the other hand, introducing extra *AKT* hindered curcumin’s ability to enhance NIS protein levels, suggesting that curcumin may enhance radioiodine sensitivity by blocking the *PI3K-AKT–mTOR* signaling pathway (Zhang et al., 2021). Other than that, Zhang et al. (2022) looked at curcumin’s effects on thyroid cancer cells from another aspect. In their study, curcumin was found to exert selective cytotoxicity on thyroid cancer cells but not normal epithelial cells and acted as an autophagy inducer through activation of *MAPK* while inhibition of *mTOR* pathways. Hyperactivation of the AKT/mTOR axis was observed in the majority of PTC samples we tested, and thyroid cancer cell lines along with cancer tissue specimens sustained a low basal autophagic activity (Zhang et al., 2022).

## Breast cancer

In breast cancer cells, a study indicates that curcumin caused a halt in the cell cycle at the G2/M phase and elevated the levels of *P21*. It also stopped the activation of *Akt/mTOR, BCL2* and boosted *BAX* while triggering caspase 3 cleavage, leading to breast cancer cell apoptosis. Overall, curcumin suppressed breast cancer cell growth and prompted G2/M phase cell cycle arrest and apoptosis, possibly by blocking *Akt/mTOR* phosphorylation and promoting mitochondrial apoptotic pathway (Hu et al., 2018). Curcumin reduced the growth of MCF-7 cells and caused cell death in a way that depended on the amount of curcumin present and the exposure time. It triggered cell death through apoptosis and raised the activities of caspase-3/9. Moreover, curcumin decreased the levels of miR-21 in MCF-7 cells by enhancing the *PTEN/Akt* pathway (Wang et al., 2017).

Besides cancer, other age-related diseases including cardiovascular diseases are also investigated in this field. For instance, curcumin, when given either orally or directly to rats or cells, helped counteract the metabolic abnormalities and increased markers of oxidative stress (such as SOD, MDA, gp91phox, and Cytochrome C) in rats with diabetes induced by streptozotocin. Additionally, it reduced apoptosis in cells (as shown by markers like *Bax/Bcl-2*, Cleaved caspase-3, and TUNEL-positive cells), while also decreasing Akt phosphorylation and increasing Foxo1 acetylation (Wang et al., 2017). By doing so, curcumin was able to relieve myocardial dysfunction, oxidative stress, and apoptosis in the hearts of diabetic rats. The use of curcumin not only increased the phosphorylation of Akt but also decreased the acetylation of Foxo1. These findings indicate that cell death was heightened in the hearts of diabetic rats, and curcumin had a part in treating diabetic heart disease by adjusting the *Sirt1-Foxo1* and *PI3K-Akt* pathways (Wang et al., 2017). A similar study declared that curcumin helped move Nrf2 into the nucleus by using the *AKT* pathway, leading to a rise in the levels of antioxidant proteins HO-1 and GCLC. As a result, this decreased the build-up of reactive oxygen species and mitochondrial harm in the heart of diabetic individuals and stopped diabetes-triggered pyroptosis (Wang et al., 2017).

## NF-κB signaling pathway

*NF-κB* is a group of protein complexes which work as rapid-acting primary transcription factors and are involved in many cellular processes including DNA transcription, cytokine generation, and cell viability (Gilmore, 2006; Gilmore and Wolenski, 2012). This signaling pathway exists in various animal cells and plays a role in how cells react to different stressors including free radicals, heavy metals, UV radiations, bacterial or viral substances, and cytokines (Gilmore, 2006; Gilmore and Wolenski, 2012). *NF-κB* stimulates the production of cytokines like IL-1, IL-6, IL-8, and TNFα, which help regulate the body’s immune system by attracting white blood cells to areas of inflammation. In addition to its involvement in the innate immune response, NF-κB also plays a role in processes like cell growth and cell death (Gilmore, 2006; Gilmore and Wolenski, 2012; Li and Verma, 2002). NF-κB contributes to aging by persistently activating inflammatory pathways, leading to cellular senescence, increased production of pro-inflammatory cytokines, and damage to cellular components, essentially accelerating the aging process when chronically activated; this includes effects like telomere shortening and impaired tissue repair mechanisms (García-García et al., 2021).

In our knowledge, there are possibly three ways which this signaling pathway can be activated through; however, two of these mechanisms are more common and well-known: the canonical way and the non-canonical way (Oeckinghaus et al., 2011; O'Dea and Hoffmann, 2009). The former way is known to be activated by the means of TNF*α* and IL-1 and microbial products. The most essential receptor which takes part in activating canonical pathway is receptor activator of NF-κB (RANK), which is a type of tumor necrosis factor receptor (TNFR) (Gilmore, 2006; Oeckinghaus et al., 2011; O'Dea and Hoffmann, 2009). Other than that, some other receptors like the toll-like microbial pattern recognition receptors (TLRs), as a member of the IL-1R family, can be involved in canonical activation. After binding to their specific ligands, a catalytic kinase named IκB kinase (IKK) complex is activated (Li and Verma, 2002; Abraham, 2000). This complex is consisting of three subunits including alpha, beta, and gamma which the IKKa and IKKb are considered to be the catalytic subunits while ΙΚΚγ or NF-kB essential modulator (NEMO) takes part in this process as a regulatory, protein sensing component (O'Dea and Hoffmann, 2009; Morgan and Liu, 2011; Williams and Gilmore, 2020). When triggered by signals, typically from outside the cell, the IKK complex adds phosphate groups to two serine sites in the IκB regulatory region (primarily IKK*β* with the aid of IKKγ subunit). When these serines are phosphorylated, the IκB proteins undergo a modification known as ubiquitination, causing them to be broken down by the proteasome inside the cell (Gilmore, 2006; O'Dea and Hoffmann, 2009; Morgan and Liu, 2011). Given the inhibitory effects of IκB on NF-κB complex, when these proteins are degraded, the p50/RelA heterodimer is free to enter the nucleus and increase the expression of some specific genes by the means of binding to κB sites of DNA (Morgan and Liu, 2011; Lawrence, 2009).

On the other hand, the non-canonical pathway is activated by some other receptors including TNF-family cytokines—lymphotoxin β (TNFSF3), CD40 ligand (CD40L and TNFSF5), B cell activating factor (BAFF and TNFSF13B), and receptor activator of NF-κB ligand (RANKL and TNFSF11) (Williams and Gilmore, 2020; Sun, 2011). Similarly, the non-canonical pathway is also starts with the activation of IKK complex; However, in this pathway, the gamma alpha subunit is responsible (not NEMO) (Abraham, 2000; Williams and Gilmore, 2020). In this pathway, NF-kB-inducing kinase (NIK) is primarily activated after receptor ligation which phosphorylates the IKK complex and thus, the activation of this complex results in the phosphorylation of two serine residues near the ankyrin repeat C-terminal IkB domain of p100, causing partial proteolysis and release of the p52/RelB complex from the IkB (Oeckinghaus et al., 2011; Sun, 2011). Besides, there is also another activation mechanism for this signaling pathway which mostly relies on oxidative stress. NF-κB is responsive to changes in Cys62 in p50 caused by oxidation, which is necessary for binding of this protein to DNA. Oxidizing conditions promote the activation and movement of NF-κB to the nucleus, while the redox-sensitive Cysteine residue hinders its ability to bind to DNA.

**Figure 3:**
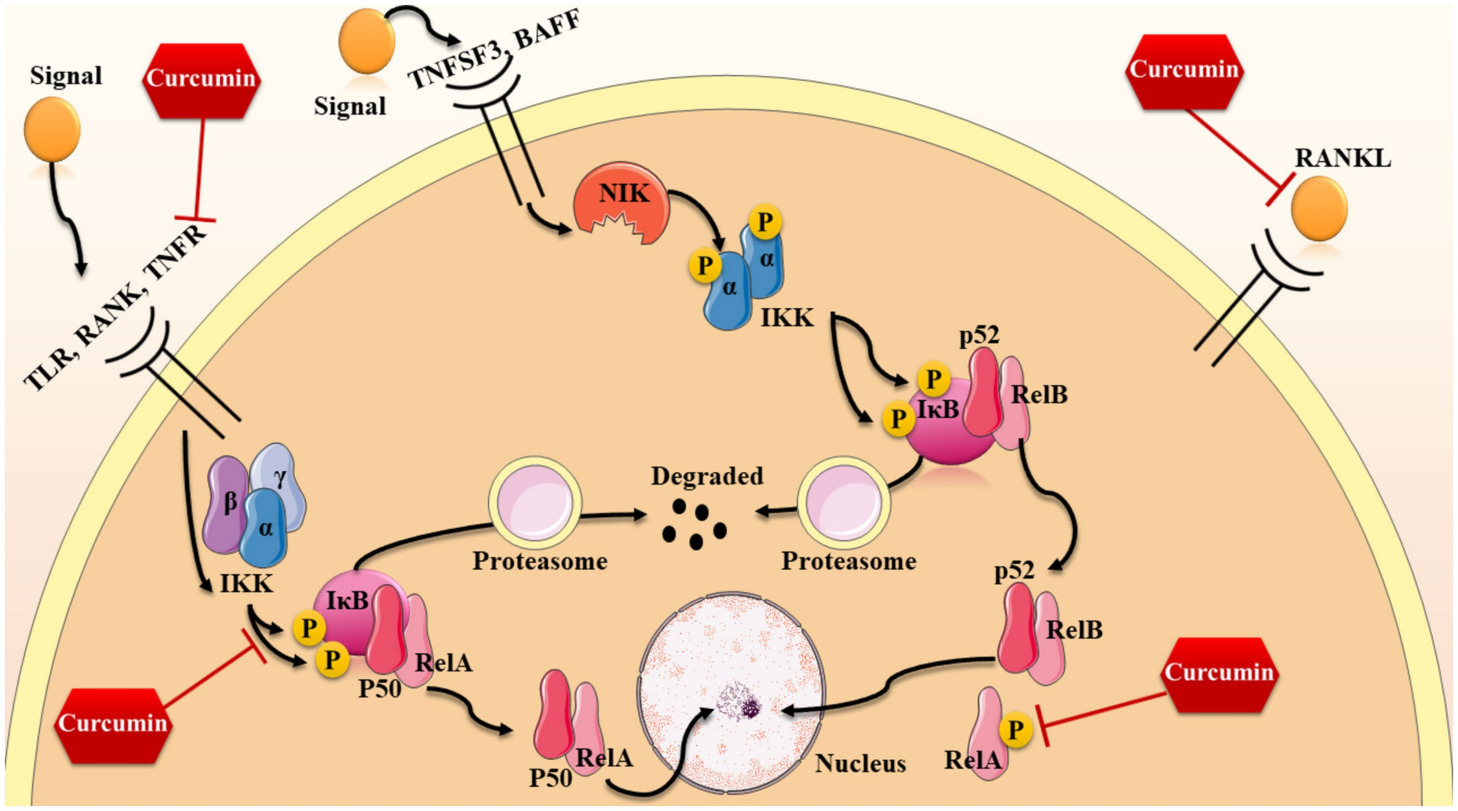
Anti-inflammation properties of curcumin.

As mentioned before, this signaling pathway has many crucial functions in regulating cellular senescence, apoptosis, cell growth, and inflammation which all are approved to be involved in the aging process (Chen et al., 2020). As an anti-inflammation and anti-oxidant agent, curcumin is also effective on several components of this pathway from its receptors to the last steps (also shown in Figure 3). For instance, RANKL (which is a ligand activating this pathway from canonical way) is affected by curcumin analogues such as ECMN909 and UBS109. These analogues are able to suppress the mentioned ligand and thereby, decreasing the preosteoclastic NF-κB-luciferase activity. These results show that curcumin and its analogues are capable of slowing down the process of osteoporosis and bone loss through enhancing osteoblastogenesis and suppressing osteoclastogenesis (Yamaguchi et al., 2012).

Moreover, it seems that the effects of curcumin analogues are not limited to this specific ligand and according this study, UBS109 is also reversing the TNF-α-induced NF-κB activation in bone tissues (Yamaguchi et al., 2012). According to recent studies, curcumin is able to make some changes in the final stages of this signaling pathway, as well. For instance, curcumin is able to significantly decrease the phosphorylation of RelA (p65) and thereby, inhibit the activation of NF-κB pathway through the canonical way; This results in the down-regulation of some inflammation-related genes including Stab1, Fdxr, Ppp3r1, and Pde8a (Lee et al., 2023). Suppressing this pathway also leads to decreasing the levels of mRNA expression of chemokine ligand 2 (Cxcl2), Cxcl10, Forkhead box O3 (FoxO3), and interleukin 6 (IL-6) in hepatic cells of aged mice (Lee et al., 2023). Besides the effects of curcumin on decreasing pro-inflammatory molecules such as IL-6 and IL-8, curcumin-induced p65 inhibition is causing a reduction in senescence-associated secretory phenotype (SASP) in mesenchymal stem cells which restores the function and the inflammation-aging signature of these cells (Mato-Basalo et al., 2021). Moreover, blocking the activity of p65 halts the spread of aging signals from mesenchymal stem cells to other cells through small extracellular vesicles containing inflammatory messages (Mato-Basalo et al., 2021).

In a recent study on heart aging, Ghorbanzadeh and colleagues compared the levels of NF-κB in aged, young, and aged rats which received curcumin. They demonstrated that treating 26–28 months’ rats 50 mg/kg of curcumin effectively increases the levels of NF-κB after 2 months; However, they did not explain the exact mechanism (Ghorbanzadeh et al., 2022). Curcumin is also able to affect the nuclear translocation of NF-κB in ischemia–reperfusion cardiac cells (Fiorillo et al., 2008). Similarly, Lv et al. (2016) also investigated the effects of a dosage of 150 mg/kg/body weight of curcumin on infarcted cardiac cells of rats and observed that in the group of participants with myocardial infarction, the NF-κBp65 expression notably rose in both the nucleus and cytoplasm, with a more significant increase in the nucleus. In comparison, the curcumin group showed significantly lower levels of NF-κBp65 expression than the myocardial infarction group (*p* < 0.01) (Lv et al., 2016). Other than myocardial infarction, hypertension, as a risk factor for cardiovascular accidents, is also affected by curcumin. Using curcumin on hypertensive rats decreases the migration of vascular smooth muscle cells via decreasing IL-1β and inactivating NF-κB (Han et al., 2019).

Regarding the role of curcumin in affecting NF-κB in neurodegenerative diseases, Alzheimer’s disease has been taken into more consideration than other diseases. One of the mechanisms by which curcumin affects this disease is through inducing the activity of the anti-oxidative Nrf2 gene which consequently leads to NF-κB suppression (Su et al., 2020). Significant improvement in learning after the suppression of microglial activation marker Iba-1, and reduction in Aβ in the brain is another beneficial impact of curcumin which is possible through NF-κB reduction (Su et al., 2020). However, it seems that TML-6 is more bioviable than curcumin and thus, using this analogue might have more efficacy in decreasing Aβ in the brain. Although the number of studies on Parkinson’s disease (PD) are so limited, a study tried to simulate PD by 6-hydroxydopamine and then used curcumin treatment to reverse this effect. They observed that curcumin is effective on the injured cells through a diversity of mechanisms which inhibiting NF-κB translocation is one of them (Wang et al., 2009).

Accumulative examinations on the effects of curcumin on stroke also shows interesting effects (Dong et al., 2014; Li F. et al., 2021; Li et al., 2017; Wu et al., 2021). Ran et al. (2021) used 150 mg/kg of curcumin intraperitoneally for 7 days on stroke-induced mice and found out that after curcumin treatment, a reduction of phosphorylation in both IκB and RelA is observed. Inhibiting NF-κB in peri-infarct areas of ischemic mice is worthwhile for enhancing white matter condition after damage, improving functional outcomes after stroke, and decreasing microglial pyroptosis (a proinflammatory programmed cell death pathway) *in vivo* and *in vitro* (Ran et al., 2021). Another study on stroke-induced mice also observed the same results and reported that administering 300 mg/kg of curcumin 30 min after stroke is beneficial for declining neurological deficits, blood brain barrier (BBB) disruption, and infarction volume through decreasing the expression of p65 (Li et al., 2016).

After detecting the mechanisms by which curcumin influences NF-κB signaling in aged cells, it is necessary to provide novel ways for enhancing the efficacy of this agent as a drug. In this regard, Tavakol et al. (2019) used Nano carriers for delivering curcumin into fibroblasts. They focused on the effect of particle size and ethanol concentration in drug delivery and found that curcumin nanoformulation that is 77 nm in size and has 3% ethanol was found to be more successful in increasing the over-expression of the β1-integrin gene, promoting anti-apoptosis in fibroblast cells (by increasing the Bcl2/Bax ratio), and reducing the expression of the *Bax* and *NF-κB* genes when compared to a nanoformulation with a smaller particle size of 50 nm (Tavakol et al., 2019). They also declared that this effect of curcumin on *NF-κB* is through inducing the expression of cell surface receptor of ß1- integrin (Tavakol et al., 2019). Another study also compared the effects of curcumin and nano-curcumin on injured cardiac cells and observed that nano-curcumin significantly decreases the amounts of p65 as well as TNF-*α*, and IL-6 in these cells. Although curcumin itself is also capable of these results but with less intensity (Sarawi et al., 2021).

Medulloblastoma is a malignant brain tumor accounting for about 20% of childhood brain tumors (Northcott et al., 2019). In the United States, about 500 children are diagnosed with medulloblastoma each year. It’s most common in children between the ages of 5 and 9, and it’s slightly more common in boys (Cotter and Hawkins, 2022). Medulloblastoma is rare in adults, accounting for only 1% of all malignant brain tumors. In the United States, only about 200 adults are diagnosed with medulloblastoma each year. It typically affects younger adults (between 20 and 40 years of age) (Franceschi et al., 2022). Spiller and colleagues found that curcumin could significantly restraint medulloblastoma progression via inhibition of NF-ĸB in a dose dependent manner (Spiller et al., 2011).

## Conclusion

Curcumin is a beta-diketone which is also known as diferuloylmethane, is an active component of turmeric (*Curcuma longa*) (Gao et al., 2012). Turmeric or *Curcuma longa* is a plant and a member of ginger family (*Zingiberaceae*), which originally is extracted from India; however, currently this plant is also grown in other countries Southeast Asia, China, and Latin America (Amalraj et al., 2017). This natural compound is examined on several cells and a diversity of diseases and is known to have a plethora of effects from its anti-inflammatory effects to free radical scavenging and inhibiting apoptosis and autophagy. Interesting effects of this polyphenol has attracted the attention of scientific community towards practical use of it as a treatment. Regarding that, age-related diseases such as atherosclerosis, cardiovascular diseases, and neurodegenerative diseases which form the majority of mortality and morbidity around the world. In this review, we discussed the effects of curcumin on oxidative stress and other mechanisms by which it can decelerate the process of aging. Furthermore, we gathered information on how curcumin regulates diverse proteins and enzymes of signaling pathways and thereby, decrease apoptosis, inflammation, senescence, and increase cell survival in aged models. All this information suggests that in spite of the high doses of curcumin administration (because of its poor absorption and rapid metabolism) no important side effects are observed for this agent which makes it a safe option for clinical use. However, more studies on this subject are required for a definite decision. There are some limitations in using curcumin as a therapeutic drug which new methods such as nano-carriers have helped fixing these problems. Using nano-formulations including nanoparticles, nano-micelles, and etc. have provided a platform for curcumin to exert its effects on its specific target cells and enhance the efficacy. The last but not least limitation in utilizing curcumin practically is that the number of human studies is not satisfying and the majority of studies have examined curcumin on animals. Given this fact and safety of curcumin we suggest that more human studies can be useful for coming to a conclusion about whether we should use this agent for treating age-related diseases or not.

## Future prospective

It seems that there are several ways by which the beneficial effects of curcumin on aged cells can be improved. According to the reviewed studies, using more bioviable analogues of this agent (such as TML-6) is one of these ways. However, more recent studies suggest the combination of nanotechnology with pharmacology and using nano-formulations for delivering curcumin to the intended site (like injured cardiac or brain cells). We suggest that designing more nano-carriers can be practical for a better use of curcumin’s potentials. Furthermore, most of studies have examined curcumin on the outcomes of aging-related diseases but we suggest that using curcumin as a preventive method might also be helpful. For instance, using curcumin before and after inducing stroke in animal models might show fabulous results and give us a new insight for seeing curcumin more than just a treatment option.
